# Antibacterial Silicon Oxide Thin Films Doped with Zinc and Copper Grown by Atmospheric Pressure Plasma Chemical Vapor Deposition

**DOI:** 10.3390/nano9020255

**Published:** 2019-02-13

**Authors:** Elisabeth Jäger, Jürgen Schmidt, Andreas Pfuch, Sebastian Spange, Oliver Beier, Nikolaus Jäger, Oliver Jantschner, Rostislav Daniel, Christian Mitterer

**Affiliations:** 1 Department of Materials Science, Montanuniversität Leoben, Franz-Josef-Straße 18, 8700 Leoben, Austria; Elisabeth.Jaeger@stud.unileoben.ac.at (E.J.); Nikolaus.Jaeger@unileoben.ac.at (N.J.); Oliver.Jantschner@stud.unileoben.ac.at (O.J.); Rostislav.Daniel@unileoben.ac.at (R.D.); 2 INNOVENT e.V. Technology Development Jena, Prüssingstraße 27B, 07745 Jena, Germany; js@innovent-jena.de (J.S.); ap@innovent-jena.de (A.P.); ss@innovent-jena.de (S.S.); ob@innovent-jena.de (O.B.)

**Keywords:** thin films, silicon oxide, copper, zinc, antibacterial films

## Abstract

Zn-doped and Cu-doped SiO_x_ films were synthesized by atmospheric pressure plasma chemical vapor deposition to study their antibacterial efficiency against Gram-negative Escherichia coli and their cytotoxic effect on the growth of mouse cells. Zn-rich and Cu-rich particles with diameters up to several microns were found to be homogeneously distributed within the SiO_x_ films. For both doping elements, bacteria are killed within the first three hours after exposure to the film surface. In contrast, mouse cells grow well on the surfaces of both film types, with a slight inhibition present only after the first day of exposure. The obtained results indicate that the films show a high potential for use as effective antibacterial surfaces for medical applications.

## 1. Introduction

Medical implants like surgical nails, screws, and plates are commonly used to immobilize fractured human bone to ensure a successful healing process. If any part of the healing sequence is altered by micro-organisms [1], the healing process may be extended dramatically. According to References [2,3], the prevalence rate of hospital-acquired infections by the colonization of the surfaces of medical implants or surgical instruments with bacteria within the Western world varies between 4% and 10%, with peak values up to 30% in intense care units. One possible solution to protect the patient from infection, sepsis, and putrefaction is to coat the implant with films containing antiseptic elements like Ag, Zn, or Cu. While these metals are well known for their antibacterial efficiency [4], medical implants and surgical instruments require multi-functional surfaces with the necessary additional wear-resistance and bio-compatibility. Thus, advanced antibacterial thin films are often based on a nanocomposite approach where the antibacterial agent is combined with a robust and inert ceramic matrix [5]. The antibacterial agent permanently needs to be available at the point of action, i.e., on the surface of an implant or instrument. This pre-condition could be met either by the immediate availability of antibacterial agents on the surface of the inert matrix or by the continuing release during its mechanical wear.

There is a plethora of published reports regarding the design and synthesis of thin films containing antibacterial agents (see References [3,5] for recent reviews). Additionally, numerous deposition techniques like atmospheric pressure plasma chemical vapor deposition (APCVD), combustion chemical vapor deposition [6], physical vapor deposition [7,8], and electrodeposition [9] have been used to deposit such antibacterial films. Recently, novel antimicrobial nanocomposites, e.g., based on halloysite nanotubes and Ag and ZnO nanoparticles [10], flax fibers with Ag and ZnO nanoparticles [11], or cellulose with Ag nanoparticles [12] have been suggested. A drawback of some of the used synthesis techniques is that they are based on the use of harmful and toxic chemicals [13], that they are not suitable to realize the required nanocomposite architecture necessary for multi-functional surfaces or that they are vacuum-based techniques resulting in high efforts and costs for production [14].

The efficiency of Ag as an antibacterial agent in the form of nanoparticles is extensively described in literature [13,15,16]. Ag may be used as antibacterial material in the form of salts, dendrimers, polymers, and metal oxide nanoparticles, and then impregnated zeolites and activated carbon materials [15,16]. The antibacterial efficiency of Ag nanoparticles is determined by the release of Ag ions, which are assumed to damage bacterial cell walls [15,16]. However, an essential drawback of Ag is that it is not inherently present in the human body, which necessitates some caution in its application [16]. 

Thus, the aim of this study is to synthesize multi-functional thin films based on a robust and bio-compatible silicon oxide (SiO_x_) matrix doped with the antiseptic elements Zn and Cu (both being trace elements in the human body) using the environmentally-friendly APCVD technique. The synthesized films are expected to provide significant antimicrobial effects against Gram-negative Escherichia coli (E. coli) and, otherwise, to ensure sufficient growth and vitality of osteoblastic mouse cells. The chemical structure and composition, the antibacterial effect, and cell growth were studied, as well as the influence of sterilization and washing procedures, with the aim to provide the first results for an optimization of the Zn and Cu content.

## 2. Materials and Methods 

APCVD combining a typical CVD process with a plasma discharge at atmospheric pressure was used to deposit the films. As substrates, pre-cleaned (isopropanol) polished B-doped 525-µm-thick Si(100) strips (10 × 20 mm^2^) as well as disks of size ϕ15 × 3 mm made of a titanium alloy of type TiAl6V4 (arithmetic surface roughness R_a_ = 515 ± 68 nm) and austenitic stainless steel (X2 CrNiMo 18 15 3, DIN 1.4441, R_a_ = 476 ± 105 nm), both supplied by Königsee Implantate, Allendorf, Germany, were used for deposition of Zn-doped and Cu-doped SiO_x_ films (in the following denominated as Zn-SiO_x_ and Cu-SiO_x_ films, respectively). As a primary precursor, evaporated hexamethyldisiloxane (HMDSO) was used to create the SiO_x_ thin film matrix. The HMDSO molecules were dissociated in the plasma discharge, mainly by collisional processes with oxygen and nitrogen molecules in the deposition chamber. The film is subsequently formed by SiO_x_ particles originating from reactions of the dissociated primary precursor with oxygen [17]. Besides the primary gaseous HMDSO precursor, solutions with zinc nitrate Zn(NO_3_)_2_ and copper nitrate Cu(NO_3_)_2_, both in a 1:1 volume mixture of isopropanol and water, were sprayed into the plasma as secondary precursors. The dissociation of the precursor molecules allowed reactions of Zn and Cu with oxygen and, consequently, formation of ZnO_x_ and CuO_x_ particles, respectively, which are subsequently incorporated into the SiO_x_ film. The variable parameters used to adjust the Zn and Cu content in the films were the flow rates of the Zn(NO_3_)_2_ and Cu(NO_3_)_2_ solutions, respectively, as summarized in Table 1.

As the plasma source for the APCVD process, a plasma jet of type BLASTER MEF (TIGRES, Marschacht, Germany) was used in combination with a modified plasma blast pipe, which enables it to feed additives as second precursors into the plasma. A mid-frequency (~40 kHz) pulsed dc plasma was ignited between two cylindrical electrodes, i.e., an inner stick electrode and the actual plasma nozzle as the grounded outer electrode. The plasma jet was accelerated using compressed air at a pressure of 6 bar. The length of the plasma jet was controlled by the applied power set to 400 W. Further details on the APCVD process are reported in previous papers [6,17,18]. The substrates were located at a distance of 10 mm below the plasma jet. All samples were moved in meandering patterns with respect to the stationary plasma jet, with a set grid spacing distance of 3 mm. To achieve a film thickness of ~100 nm, 8 runs during the deposition process were realized.

While the silicon substrates were used for film characterization by scanning electron microscopy (SEM), energy dispersive X-ray spectroscopy (EDX), atomic force microscopy (AFM), and X-ray photoelectron spectroscopy (XPS), the TiAl6V4 and the austenitic stainless steel disks were used for the application-oriented tests. In this case, TiAl6V4 represents a typical implant material, while austenitic stainless steels are widely used for surgery instruments. Both film thickness and arithmetic surface roughness R_a_ were measured using a stylus contact profilometer (Dektak 3 ST). The film surface was investigated using a Carl Zeiss SUPRA 55 VP SEM using acceleration voltages of 5 and 15 kV and working distances of 4.4 and 10.4 mm. The size distribution of particles formed on the film surface and visible in the SEM micrographs was statistically analyzed by the free software ImageJ [19]. In addition, the surface topography of the films was characterized using a SIS ULTRAObjective AFM in the non-contact mode. EDX (Quantax with Si(Li)-detector, Röntec) and XPS (Theta Probe, Thermo VG Scientific) determined the chemical composition of the films. The oxidation states of Zn, Cu, and Si were identified by XPS using an X-ray excitation energy of 1468.68 eV (Al Kα line). The 2p core level spectra were used for determining the respective metal oxides. No thermal treatment or sputter cleaning of the surfaces was done prior to the XPS measurements. As energy calibration, the binding energy of the C 1s level (285 eV), taken from the carbon contamination, was used. To probe the resistance of the films against abrasive wear and, consequentially, to evaluate the residual antiseptic effect of the films, a washability test (Elcometer, Simex) in distilled water was performed using nylon brushes and a stainless steel holder, according to the ASTM D2486 standard. The number of washing cycles varied from 1000 to 10,000.

The fluorescein diacetate/GelRed live/dead staining assay was used to study the cytotoxic effect of the films on osteoblastic mouse cells of type MC 3T3-E1. The cells were cultured at 37 °C under 5% CO_2_ atmosphere in Dulbecco’s modified Eagle’s medium with 10% fetal calf serum, which was supplemented with 50 U/mL penicillin and 50 µg/mL streptomycin (Biochrom, Berlin, Germany). For the test, the cells were brought into direct contact with the film surface in a density of ~12,500 cells/cm^2^. After one, three, and seven days of incubation, the live/dead staining assay was performed. The cell coloration micrographs were taken using a Carl Zeiss Axiotech fluorescence microscope.

The antibacterial effect of the films was probed using a Gram-negative E. coli strain. The BacTiter-Glo microbial cell assay (BTG, Promega, Mannheim, Germany) was used to detect bacterial injury on the basis of the intracellular adenosine triphosphate (ATP) content of the cells. For the test, E. coli bacteria were brought into direct contact with the film surface. Each sample was immersed in 0.5 mL of the diluted E. coli working suspension for 3 h. The developed luminescence signals were recorded in a microplate reader (Genios Pro, Tecan), which enables luminescence detection using an integration time of 400 ms. An antibacterial effect was detected if the BTG test showed a decrease of luminescence in comparison to the unaffected control samples.

Sterilization tests were done under hospital cleaning and sterilization conditions (Thüringen-Kliniken “Georgius Agricola”, Germany) to evaluate the antibacterial effect of the films after such treatments. The cleaning step over 50 min was realized by a cleaning, disinfection, and drying machine (Belimed WD380) with a washing temperature of 93 °C and a drying temperature of 110 °C. The subsequent sterilization step of 5 min was performed by using a steam-sterilizer (MMM Selectomat), where the temperature ranged from 134 to 137 °C and the pressure was between 3034 and 3320 mbar. Subsequently, the BTG test was performed on the film surface.

## 3. Results

### 3.1. Film Morphology and Chemical Composition

**Figure 1:**
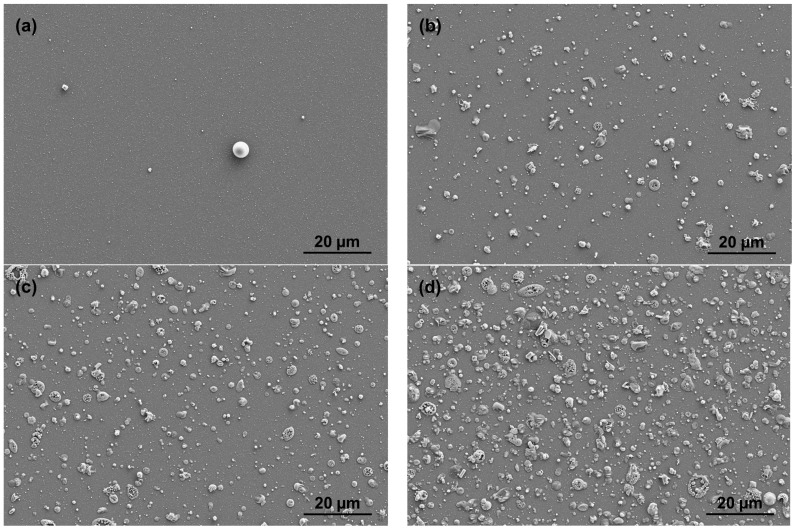
SEM micrographs of the surface topography of Zn-SiO_x_ films grown on Si with secondary precursor flow rates of (**a**) 0, (**b**) 25, (**c**) 50, and (**d**) 100 µL/min.

**Figure 2:**
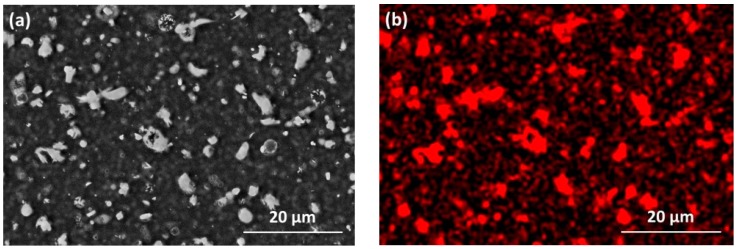
(**a**) SEM micrograph of the surface topography and (**b**) the corresponding Zn EDX mapping for a Zn-SiO_x_ film grown on Si with a secondary precursor flow rate of 100 µL/min.

All films were optically transparent and showed no evidence of delamination. For the Zn-SiO_x_ films, the series of SEM surface topology micrographs presented in Figure 1 show that only a low number of small particles is visible on the surface of SiO_x_ films deposited without a secondary precursor, whereas both the density and size of these particles increase significantly for the increasing secondary precursor flow rate. The SEM micrograph and the Zn elemental map obtained by EDX depicted in Figure 2 clearly indicate that the micron-sized particles visible in Figure 1b–d are rich in Zn. Besides these Zn-rich particles, a lower Zn concentration was detected to be homogeneously distributed throughout the film.

**Figure 3:**
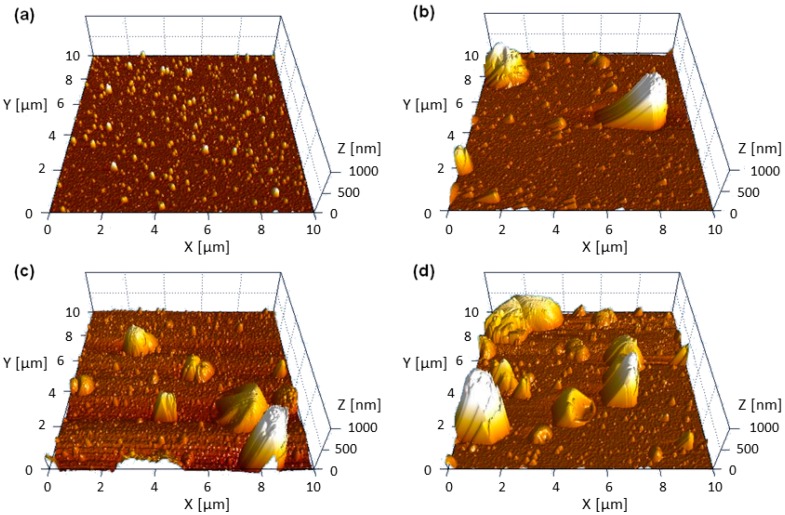
AFM surface topography images of Zn-SiO_x_ films grown on Si with secondary precursor flow rates of (**a**) 0, (**b**) 25, (**c**) 50, and (**d**) 100 µL/min.

Both austenitic stainless steel and TiAl6V4 substrates coated with Zn-SiO_x_ and Cu-SiO_x_ films exhibit a surface roughness R_a_ determined using a stylus contact profilometer in the range of 480 to 1120 nm. The upper values have been obtained for high flow rates of the secondary precursor (see Table 1 and Figure 1) and they are considerably higher than those of the uncoated reference samples (see Section 2) or for samples coated with a pure SiO_x_ film (R_a_ = 340–540 nm). They also significantly exceed the film thickness of ~100 nm. This is attributed to the formation of the particles visible in Figure 1 and Figure 2. Figure 3 represents a series of AFM surface topography images of Zn-SiO_x_ films deposited at different flow rates of the secondary precursor, which shows that the un-doped SiO_x_ film is quite smooth, whereas major particles in the Zn-SiO_x_ films protrude up to 1 µm above the film surface.

**Figure 4:**
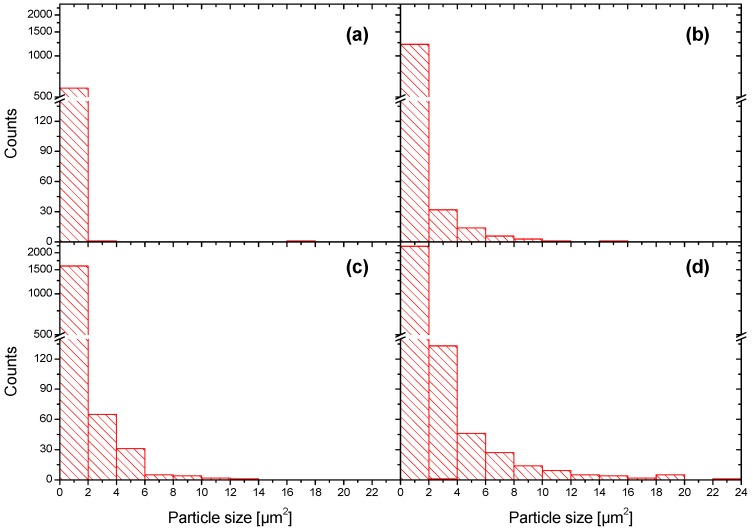
Particle size distribution determined by statistical analysis using the SEM micrographs shown in Figure 1 for Zn-SiO_x_ films grown on Si with secondary precursor flow rates of (**a**) 0, (**b**) 25, (**c**) 50, and (**d**) 100 µL/min.

The particle size distribution on the surface of Zn-SiO_x_ films grown on Si was statistically analyzed using the SEM micrographs presented in Figure 1. Figure 4 indicates that the vast majority of particles visible on the surface of an un-doped SiO_x_ film occupies an area of less than 2 µm^2^. A more detailed analysis and Figure 3a show a particle diameter of up to 200 to 300 nm. In contrast, these small particles characteristic for the un-doped SiO_x_ film are still present for an increasing flow rate of the secondary precursor, whereas the fraction of larger and, according to Figure 2b, Zn-rich particles increases considerably. The maximum particle size observed on Zn-SiO_x_ films increases from 12 to 14 µm^2^ (corresponding to a diameter of 3.9 to 4.2 µm of an equivalent circular particle) to 22–24 µm^2^ (5.3–5.5 µm diameter) for increasing secondary precursor flow rates (see Figure 4). 

The formation of slightly smaller and less frequently appearing Cu-rich particles on the surface of the Cu-SiO_x_ films indicates the same growth mechanism of the particles such as in the case of Zn-SiO_x_. The formation of these particles corresponds well to earlier findings on SiO_x_ film with embedded Ag particles grown by APCVD [16]. To sum up, all formed films can be described as composites with Zn-rich or Cu-rich particles, which are characterized by a diameter range of up to several microns and embedded into a SiO_x_ film formed by particles of up to 200–300 nm in diameter.

**Figure 5:**
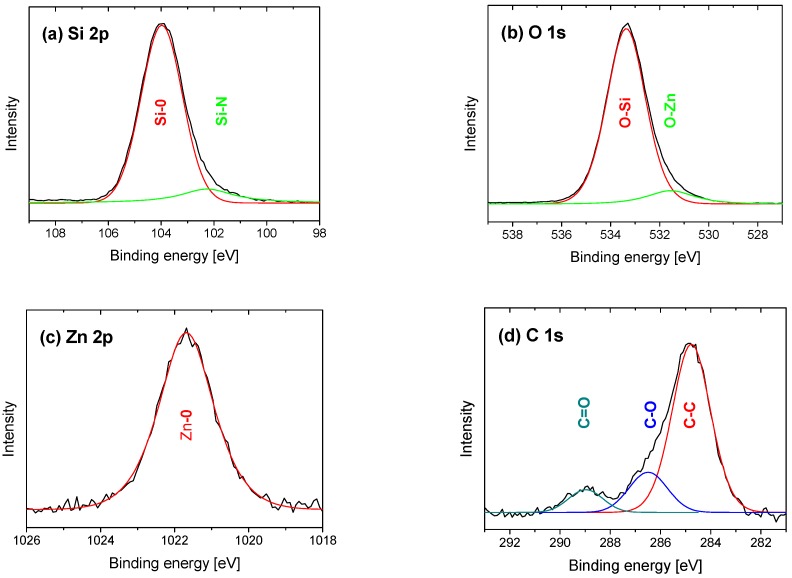
XPS spectra for the (**a**) Si 2p, (**b**) O 1s, (**c**) Zn 2p, and (**d**) C 1s orbitals of the Zn-SiO_x_ film grown onto Si with a secondary precursor flow rate of 100 µL/min.

Figure 5 shows the XPS spectra of the Zn-SiO_x_ film grown at the highest flow rate of the secondary precursor of 100 µL/min. Peak fit analysis was used to separate the spectra into individual components. The peaks in the Si 2p spectrum shown in Figure 5a located at 103.9 and 102.2 eV correspond to Si-O and Si-N bonds, respectively, which is in good agreement with literature [20]. The Si-N bonds originate from the small amount of nitrogen incorporated in the films from fractions that are not fully dissipated as a secondary precursor. The two peak positions in the O 1s spectrum in Figure 5b located at 533.3 and 531.5 eV correspond to O-Si and O-Zn bonds, which agree well with the Si-O bonds at 103.0 eV in Figure 5a and the Zn-O bonds at 1021.7 eV in Figure 5c. The observed binding energies of 531.5 eV and 1021.7 eV correspond well to those reported for stoichiometric ZnO nanoparticles [21], which indicate a composition close to ZnO for the particles incorporated in the Zn-SiO_x_ films. In addition, the absence of Si-Si and Zn-Zn bonds in the surface near the region indicates full oxidation of the film species (besides the minor nitride fraction mentioned above), which is due to the much higher enthalpy of formation of the oxide components with respect to the metallic bonding [20]. The C 1s spectrum in Figure 5d indicates carbon surface contamination at 284.5 eV, with the additional peaks between 286 and 290 eV identified as C and O containing species. Minor bulk carbon contaminations, as visible by the slightly enhanced XPS signal at 99.85 eV in Figure 5a, correspond to Si-C bonds, and, at 282.5 eV in Figure 5b, correspond to C-Si bonds [22], which could originate from the HMDSO precursor. The Zn content of the doped films determined by EDX of 2.3 at.% at a secondary precursor flow rate of 100 µL/min is in good agreement with the value obtained by XPS of 1.3 at.% and decreases with a reducing flow rate, which is reflected by a reduced Zn 2p signal.

**Figure 6:**
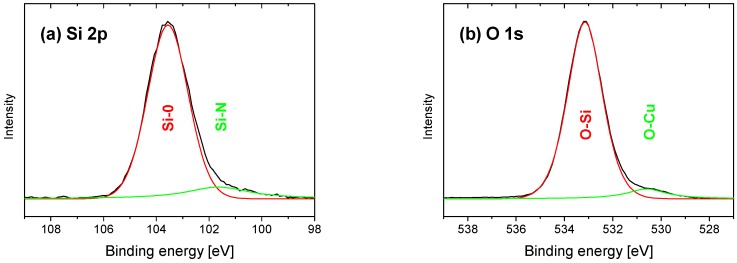

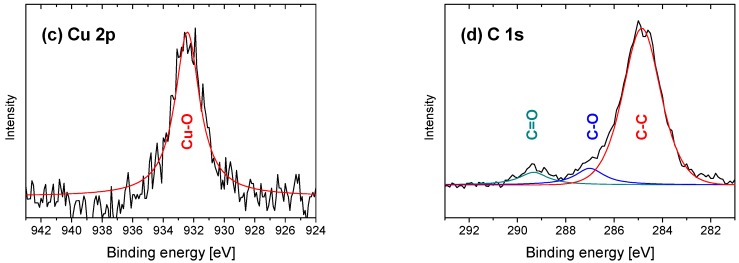
XPS spectra for the (**a**) Si 2p, (**b**) O 1s, (**c**) Cu 2p, and (**d**) C 1s orbitals of the Cu-SiO_x_ film grown onto Si with a secondary precursor flow rate of 50 µL/min.

Figure 6 shows the XPS spectra of the Cu-SiO_x_ film grown at the highest secondary precursor flow rate of 50 µL/min. The spectra were again separated into their individual components. In analogy to the Zn-SiO_x_ films, the peaks in the Si 2p spectrum in Figure 6a located at 103.5 and 101.6 eV correspond to Si-O and Si-N bonds, respectively, where the Si-N bonds stem from the not fully dissipated secondary precursor. The two peak positions in the O 1s spectrum in Figure 6b located at 533.1 and 530.6 eV correspond to O-Si and O-Cu bonds, which align with the Si 2p and the Cu 2p spectra in Figure 6a,c. The differences in binding energies of Cu and O in CuO and Cu_2_O reported in Reference [23] lead to the conclusion that Cu_2_O is present within the Cu-SiO_x_ films. In addition, within this scenario, the absence of the Si-Si and Cu-Cu bonds indicates full oxidation of the film in the surface near region. This result is in good agreement with the findings of Maroie et al. [24]. The contamination of the Cu-SiO_x_ film by carbon-containing species is similar to the Zn-SiO_x_ films, which is indicated by the C 1s spectrum in Figure 6d. Due to a generally lower secondary precursor flow rate during deposition of the Cu-SiO_x_ films, the Cu content was systematically lower with respect to the Zn content in the Zn-SiO_x_ films. The maximum concentration of Cu determined by EDX and XPS was, thus, only ~0.2 at.% at the maximum flow rate of the secondary precursor of 50 µL/min.

### 3.2. Cytotoxic Effect

**Figure 7:**
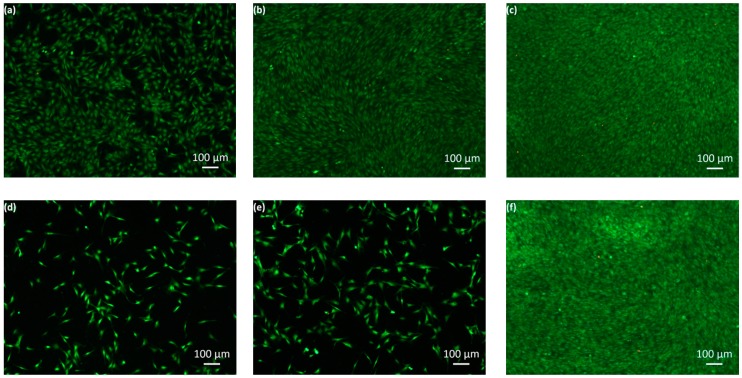

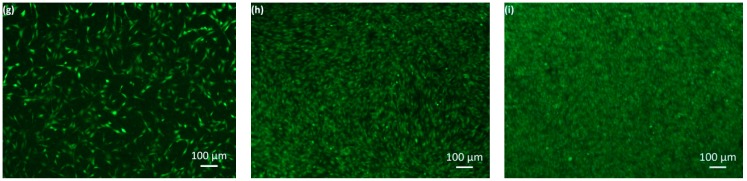
Representative live/dead staining images for austenitic stainless steel disks coated with (**a**–**c**) an un-doped SiO_x_ film, (**d**–**f**) a Zn-SiO_x_ film grown at a secondary precursor flow rate of 100 µL/min, and (**g**–**i**) a Cu-SiO_x_ film grown at 50 µL/min on the first (**a**,**d**,**g**), third (**b**,**e**,**h**), and seventh evaluation day (**c**,**f**,**i**).

The development of the number of cells grown on the surfaces of the un-doped SiO_x_ as well as Zn-SiO_x_ and Cu-SiO_x_ films during exposure to osteoblastic mouse cells of type MC 3T3-E1 characterized by the live/dead staining assay is visualized in Figure 7 for those films produced at the highest flow rates of the respective secondary precursor on austenitic stainless steel substrates. A dispersed green fluorescent cell floor (i.e., a negligible number of dead cells) indicates the spread cell formation after staining the samples post one day of incubation (Figure 7a,d,g). For comparison, the uncoated reference samples showed the same dispersed green fluorescent cell floor, which does not have a significant cytotoxic effect. The micrographs taken after one (Figure 7a,d,g) and three days of exposure (Figure 7b,e,h) indicate no cytotoxic effect but a certain inhibition in cell growth, which is more pronounced for the Zn-SiO_x_ compared to the Cu-SiO_x_ film. A dramatic increase of the cell density on all film surfaces after seven days of the test results in an unhampered cell growth (see Figure 7c,f,i). After these seven days, the development of the cell growth in the case of SiO_x_ is similar to that observed for the doped films, which shows that the addition of Zn and Cu does not disturb cell growth.

### 3.3. Antibacterial Activity

**Figure 8:**
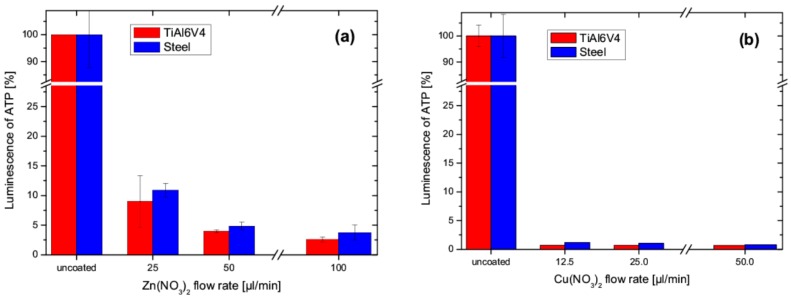
Influence of the flow rate used for (**a**) Zn-SiO_x_ films grown with a secondary precursor concentration of 5 wt.% Zn(NO_3_)_2_ and (**b**) Cu-SiO_x_ films grown with a secondary precursor concentration of 2.5 wt.% Cu(NO_3_)_2_ on detectable luminescence of ATP compared to uncoated austenitic stainless steel and TiAl6V4 reference samples.

Figure 8a shows the antibacterial activity of the Zn-SiO_x_ films, expressed by the luminescence of ATP with respect to the uncoated reference austenitic stainless steel and TiAl6V4 samples for an increasing secondary precursor flow rate. In an earlier study, no significant antibacterial effect of the un-doped SiO_x_ film, as expressed by the luminescence of ATP, was determined [17]. A significant decrease of luminescence is visible for the increasing Zn(NO_3_)_2_ flow rate, where a flow rate of 25 µL/min was sufficient for an unambiguous antibacterial activity. Compared to Zn-SiO_x_, the Cu-SiO_x_ system showed a considerably more pronounced antibacterial effect (see Figure 8b), irrespective of the film composition and the used substrate, even for the reduced Cu(NO_3_)_2_ concentration of 2.5 wt.% obtained for the secondary precursor flow rate of 50 µL/min. The luminescence of ATP decreased to 1% for all flow rates investigated. Thus, it can be concluded that an antibacterial effect is evident for Cu-SiO_x_ films grown with a much lower secondary precursor flow ratio compared to the Zn-SiO_x_ films (see Section 3.1 and compare Figure 8a,b).

**Figure 9:**
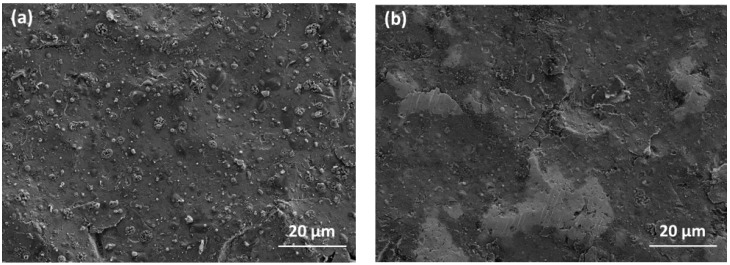
SEM micrographs of the surface of a Zn-SiO_x_ coated austenitic stainless steel surface (secondary precursor concentration of 5 wt.% Zn(NO_3_)_2_, flow rate of 100 µL/min). (**a**) As-deposited state and (**b**) after 1000 washing cycles.

Degradation of the film surface during the washability test results in a modification of the film and substrate surface topography, as shown in Figure 9 for the Zn-SiO_x_ film in the as-deposited state and after 1000 washing cycles. After these washing cycles, the film surface appears smoother (R_a_ = 500 nm, compare Figure 9b) than the pristine reference film sample (R_a_ = 1120 nm, compare Figure 9a). Most of the micron-sized ZnO_x_ and CuO_x_ particles and agglomerates formed on the film surface during the APCVD process were scrubbed from the surface during the washing procedure (as shown in Figure 9b by the significant reduction of the particle number), which reduces the fraction of antibacterial agents on the surface (the highest Zn concentration was detected in the particles, see Figure 2). In good agreement with this finding, for both the Zn-SiO_x_ and the Cu-SiO_x_ film, the content of Zn and Cu after 1000 washing cycles was close to the detection limit of EDX, which prevents providing reliable numbers for the loss of these antibacterial agents. However, it should be noted that another contribution to the surface smoothening observed during the washing procedure is provided by plastic surface deformation of the substrate material, as indicated by large flattened areas appearing bright in Figure 9b.

**Figure 10:**
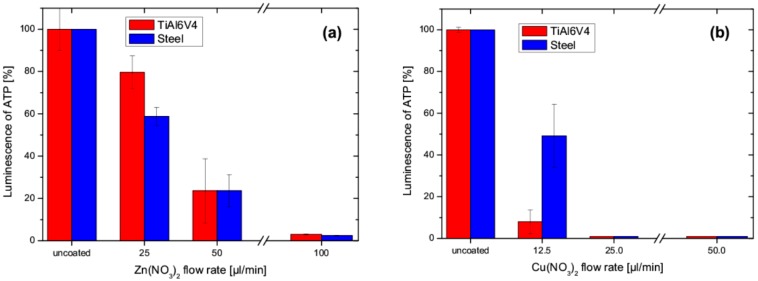
Influence of the flow rate used for (**a**) Zn-SiO_x_ films grown with a secondary precursor concentration of 5 wt.% Zn(NO_3_)_2_ and (**b**) Cu-SiO_x_ films grown with a secondary precursor concentration of 2.5 wt.% Cu(NO_3_)_2_ on detectable luminescence of ATP after 1000 washing cycles. Uncoated austenitic stainless steel and TiAl6V4 samples are included as references.

The mechanical loads imposed to the film surface during the washability test affect their antibacterial ability. Figure 10a indicates a strong increase in the luminescence signals of ATP for the Zn-SiO_x_ samples coated with secondary precursor flow rates of 25 and 50 µL/min Zn(NO_3_)_2_ after 1000 washing cycles compared to the pristine film surfaces, which reach higher values and, thus, reduced or even nearly lost antibacterial ability (compare Figure 8a and Figure 10a). However, for the highest precursor flow rate, an even more pronounced antibacterial ability compared to the pristine surface condition was obtained, which is most probably related to exposure of additional fresh ZnO_x_ nanoparticles, originally hidden within the SiO_x_ matrix, by the washing procedure.

The luminescence of ATP of the surface of the Cu-SiO_x_ films after 1000 washing cycles is presented in Figure 10b. It reaches higher values than those of the pristine film surfaces (compare Figure 8a and Figure 8b), which is most pronounced for the film with the lowest Cu fraction. With an increasing secondary precursor flow ratio, the difference in the antibacterial ability observed for the pristine surface and for the surface after 1000 washing cycles decreases. After further increasing the number of washing cycles up to 10,000, no reliable antibacterial effect could be detected anymore for both the Zn-doped and Cu-doped films.

**Figure 11:**
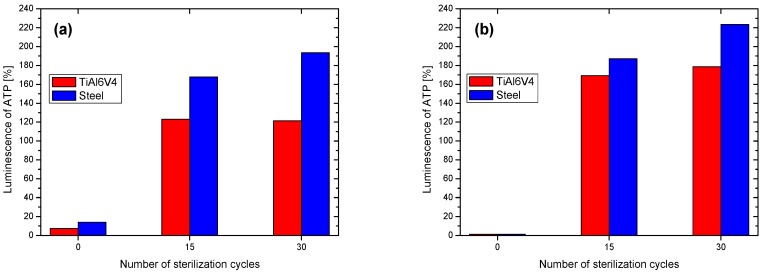
Luminescence of ATP of (**a**) Zn-SiO_x_ (Zn(NO_3_)_2_ flow rate of 100 µL/min) and (**b**) Cu-SiO_x_ films (Cu(NO_3_)_2_ flow rate of 50 µL/min) after 0, 15, and 30 sterilization cycles.

During sterilization, the film surface is exposed to heat, pressure, and chemicals like ethanol, which can be assumed to affect its antibacterial ability. The effect of the sterilization process on the antibacterial behavior of both the Zn-SiO_x_ and the Cu-SiO_x_ films deposited using secondary precursor flow rates of 100 and 50 µL/min, respectively, is summarized in Figure 11. In both cases, the luminescence of ATP after 15 and 30 sterilization cycles increases significantly when compared to the pristine film. This increase is more severe for the Cu-SiO_x_ than for the Zn-SiO_x_ films, and more pronounced for the austenitic stainless steel than for the TiAl6V4 substrates.

## 4. Discussion

Ag-doped SiO_x_ was demonstrated earlier to be a promising candidate for antibacterial films for medical applications [6,17]. Alternatively, antiseptic metals like Zn or Cu, which are present as essential trace elements in the human body, could reduce the risks of undesired reactions when applied to the surfaces of medical implants or surgical instruments. According to the present knowledge, contact killing with Cu ions is assumed to proceed by successive cell membrane damage, Cu influx into the cells, oxidative damage, cell death, and degradation of the deoxyribonucleic acid [25]. Zn ions are potential inhibitors of catabolism and O_2_ metabolism. They inhibit hydrogen peroxide production nearly completely, but also enhance killing by peroxide added to the cells [26,27].

The Zn-SiO_x_ and Cu-SiO_x_ films synthesized by APCVD within this research study exhibit a high density of particles on the surface, which is related to the deposition process [17]. These particles and agglomerates with sizes up to several microns are rich in Zn or Cu, respectively, and their chemical state corresponds to ZnO or Cu_2_O. Next to the SiO_x_ matrix, they represent the dominating part of the film, especially on top of the film surface (see Figure 1, Figure 2 and Figure 3). A higher secondary precursor flow rate leads to a higher particle size and density, which correlates well with a higher Cu and Zn fraction on the film surface. The principal formation of such large particles in the gas phase is related to the kinetics of particle condensation/nucleation and growth, which is not balanced by high mass transport rates during the deposition process, and was also found for adding Ag particles to an SiO_x_ film grown by APCVD [17].

Osteoblastic mouse cells of type MC 3T3-E1 grow well on both Zn-doped and Cu-doped SiO_x_ films with a slight inhibition effect present only after the first day of exposure and limited inhibition or no inhibition later (see Figure 7). Thus, no cytotoxic effect was observed. This behavior is independent of the applied secondary precursor flow rates and substrates. The slight inhibition of cell growth might be related to the pronounced release of the antiseptic Zn or Cu ions or even metal oxide nanoparticles, respectively, from the pristine film surface, which results in high efficiency for reducing bacteria, but also affects the growth of osteoblastic cells. Later-on, the film surface can be assumed to be shielded by dead cells, which suppresses the release of antiseptic ions and consequently promotes cell growth. The more pronounced inhibition of cell growth observed for the Zn-SiO_x_ compared to the Cu-SiO_x_ film is most probably related to the significantly higher Zn content of up to ~2.3 at.% compared to ~0.2 at.% Cu derived for the highest secondary precursor flow rates. A similar effect was also observed in experiments with Ag [8,28]. 

While E. coli bacteria are exposed to the film surface, they are in direct contact with metal ions released in a humid environment or with oxide nanoparticles. The Cu-doped films showed a more pronounced antibacterial effect compared to Zn-SiO_x_, despite the lower Cu concentration within the films, given by the lower secondary precursor concentration and flow rates used during film deposition (see Table 1). This might be attributed to the significantly lower ionization energy of Cu (7.7264 eV) compared to Zn (9.3941 eV), which results in a more pronounced release of Cu ions. For comparison, the ionization energy of Ag (7.5762 eV) is even slightly lower than that of Cu, which results in its superior antibacterial efficiency [29]. The antibacterial efficiency of Zn in the E. coli suspension used in this study can be assumed to be lower than in dry environments, due its cell drying properties. Another explanation of the antibacterial ability of the films could be based on the contact to metal oxide nanoparticles, which is present in the films investigated within this study. In particular, CuO nanoparticles have been reported to display the highest potency in causing cell death when compared to other metal oxides like TiO_2_, ZnO, Fe_3_O_3_, or Fe_2_O_3_ [30].

The washability test leads to a significant change of the surface topography of the films, since a major fraction of the ZnO_x_ and CuO_x_ particles have been mechanically removed after 1000 washing cycles. Beier et al. reported that, during the first ten washing cycles, a high loss of antiseptic Ag from the film surface was observed. After this burst effect, only a slightly further decreasing fraction of released Ag was found in up to 10,000 washing cycles [17]. Taking into account that most of the micron-sized ZnO_x_ and CuO_x_ particles are scrubbed from the surface after 1000 washing cycles (see Figure 9), the concentration of Cu and Zn, respectively, has to be assumed to decrease dramatically in the same manner like in the Ag particles stated above. Nevertheless, the fraction of metal ions or metal oxide nanoparticles released from the film surface for the highest secondary precursor flow rates is still sufficient for reducing the population of E. coli bacteria (see Figure 10). 

Beier et al. reported that no antibacterial activity of Ag-containing samples re-tested by repetitive interaction with an E. coli suspension and subsequent sterilization was found [17]. This is related to the more stringent procedure applied (exposure to E. coli, sterilization with ethanol for 1 h, washing with distilled water and ethanol, drying), which promotes the fast depletion of ions from the film surface, compared to the washing cycles applied in this work. Another possible reason could be the sealing of the film surface by the sterilization procedure, which hinders the release of Ag ions [17]. Airey and Verran reported a decrease of the contact killing efficiency of Cu after the second soiling/cleaning cycle [31]. However, in a different study, Cu surfaces have been mentioned to remain active when soiled [32]. While there are ambiguous findings on the degradation of the antibacterial properties of Cu in the literature, the present understanding of the mechanism of contact killing leads to the conclusion that a clean surface, without significant oxide scales and free of wax or other films, easily releases the antibacterial agent and will always be active in contact killing [6,25]. The antibacterial activity has been reported to be lost for Ag-doped SiO_x_ films after annealing at 500 °C in ambient air. Ag, as a noble metal, was reported to be incorporated in metallic form into the SiO_x_ matrix [6], which forms the thermally unstable silver sulfite in ambient industrial atmospheres containing sulfur. In contrast, ZnO_x_ and CuO_x_ should be able to survive the thermal exposure during the sterilization process. Thus, it can be concluded that the films investigated within this study are primarily degraded by soil and by washing procedures, which explains why they lost their antibacterial functionality after the performed sterilization tests (see Figure 11).

## 5. Conclusions

SiO_x_ films with different fractions of the doping elements Zn and Cu, respectively, were synthesized by atmospheric pressure plasma chemical vapor deposition and evaluated with respect to their antibacterial and cytotoxic effects. The formed films represent composites with ZnO and Cu_2_O particles, with diameters up to several microns, embedded into a SiO_x_ matrix. The best antibacterial ability was obtained for the Cu-containing film with the highest Cu content of 0.2 at.%. While Gram-negative Escherichia coli bacteria exposed to the film surface are killed within three hours, mouse cell growth is inhibited only after the first day of exposure, with limited or no inhibition later. Zn-doped SiO_x_ films with slightly less pronounced antibacterial properties are obtained for a Zn concentration of 2.3 at.%. While both Zn-doped and Cu-doped SiO_x_ films are mechanically stable and survive 1000 washing cycles with only slightly affected antibacterial behavior for sufficient metal contents, the sterilization procedure using standard hospital cleaning and steam sterilization conditions results in rapid deterioration of the antibacterial efficiency. The formed composites based on a robust and bio-compatible SiO_x_ matrix with added Cu_2_O and ZnO particles grown by the environmentally-friendly atmospheric plasma chemical vapor deposition technique represent promising candidates for antibacterial thin films meeting the requirement of multi-functional surfaces for medical implants and surgical instruments.

## Figures and Tables

**Table 1:** Secondary precursor conditions used for the APCVD process.

Secondary Precursor	Zn(NO_3_)_2_	Cu(NO_3_)_2_
Concentration [wt.%]	5	2.5
Isopropanol:water volume ratio	1:1	1:1
Flow rate [µL/min]	25; 50; 100	12.5; 25; 50
